# Dot1l Regulates the Spontaneous Bone Regeneration of Periosteum-Derived Stem Cells by Regulating Chac1 Expression

**DOI:** 10.1155/sci/1508850

**Published:** 2025-07-09

**Authors:** Taoran Jiang, Bin Fang, Zheyuan Yu, Dejun Cao

**Affiliations:** Department of Plastic and Reconstructive Surgery, Shanghai 9th People's Hospital, Shanghai Jiao Tong University School of Medicine, Shanghai 200011, China

**Keywords:** Chac1, Dot1l, maxillofacial bone defect, osteogenic differentiation, PDSCs

## Abstract

**Background:** The periosteum plays an indispensable role in bone repair, and promoting osteogenic differentiation of periosteum-derived stem cells (PDSCs) is one of the most effective strategies for enhancing spontaneous bone regeneration in maxillofacial bone defects.

**Methods:** We established a rat model of mandibular defects with preserved periosteum to explore its bone regeneration capacity and the potential mechanisms of PDSC activation and osteogenic differentiation.

**Results:** Significant bone regeneration was observed in rats with preserved periosteum after mandibular defects. To explore the underlying mechanisms, PDSCs were isolated from the periosteum of rat mandibles, and the stem cell markers CD90 and CD44 was highly expressed in these PDSCs. Further, RNA-seq, RT-qPCR, and Kyoto Encyclopedia of Genes and Genomes (KEGG) functional analyses revealed significantly reduced expression of the Dot1l gene, and the Notch pathway was significantly enriched in the PDSCs of the model group. Osteogenic assays demonstrated that the overexpression of Dot1l significantly inhibited the alkaline phosphatase (ALP) activity, calcium deposition, and the expression of osteogenic-related genes (such as RUNX2, OSX, ALP, and OCN) in PDSCs. Additionally, Dot1l significantly affects the Notch signaling pathway in the Gene Ontology (GO) pathways, and significantly downregulates the expression of Chac1 within it. Further, Dot1l inhibited ALP activity, calcium deposition, and the expression of osteogenic-related genes in PDSCs by downregulating Chac1 expression.

**Conclusions:** Our study suggests that mandibular defects can induce the activation of PDSCs and inhibit the expression of Dot1l, potentially affecting the Notch signaling pathway. Targeting the Dot1l/Chac1 pathway to regulate the osteogenic differentiation of PDSCs lays a solid foundation for periosteum-based maxillofacial bone regeneration.

## 1. Introduction

Maxillofacial bone defects are common and challenging clinical problems faced by oral and maxillofacial surgeons. Most of these defects are usually caused by congenital diseases or acquired conditions such as trauma, infection, tumor, osteomyelitis, and other types of surgical debridement [1]. The jaw is one of the most important structures in the maxillofacial region. Jaw defects can impact various physiological functions, such as chewing and swallowing, seriously affect the quality of life and mental health, and may even pose a risk to life. Autogenous bone graft is the gold standard for jaw defect repair. Although it is the preferred choice, it has limitations, such as the donor site damage and limited materials availability. While allogeneic bone and artificial bone are relatively simple to operate, but issues such as immune rejection and poor fusion also limit the clinical application [2–5]. Therefore, it is fortunate that stem cells play a crucial role in bone-tissue engineering. Due to its multifunctional differentiation, self-renewal ability, extensive sources, and other factors, it has become an ideal seed cell in the field of bone regeneration, and has great potential to promote bone repair and regeneration [6–8].

The repair and regeneration of bone defects cannot be separated from two key ossification processes, including endochondral ossification and intramembranous ossification. Endochondral ossification is the gradual transformation of cartilage into bone tissue, which mainly occurs during the development of long bones. Intramembranous ossification is the formation of bone directly on fibrous membranes by osteoblasts, which usually plays an important role in the development of flat bones [9]. The maxillofacial bone is classified as flat bone. Intramembranous ossification in the repair process of maxillofacial bone is the process of generating bone tissue directly in the connective tissue membrane, which is crucial for the repair of jaw defects. This process relies primarily on the bone-making ability of stem cells [10]. Bone marrow mesenchymal stem cells (BMSCs) have the ability to self-renew and repair, and have a significant impact in bone regenerative cell therapy. Unfortunately, however, BMSCs are not the core cells involved in endogenous bone repair. In addition, maxillofacial bone lacks bone marrow, making it difficult to recruit enough BMSCs [11, 12]. In contrast, periosteal stem cells have a high rate of clonogenesis and cell proliferation, and they are at the top of the differentiation hierarchy. Periosteum-derived stem cells (PDSCs) still exhibit strong self-renewal and differentiation capabilities [11, 13, 14]. Studies have shown that periosteum, a thin layer of connective tissue covering the bone surface with a rich blood supply and extensive nerve distribution, is a promising source of stem cells for endogenous bone healing [14, 15]. Studies have also shown that bone defects with preserved periosteum have better bone healing outcomes than those with removed periosteum [16]. Therefore, PDSCs play an important role in bone repair. However, the specific mechanism of their activation is still unclear.

Dot1l, an H3K79 methyltransferase, regulates developmental processes including embryonic development [17], erythropoiesis [18], cardiac differentiation [19], and cartilage homeostasis [20]. Specifically in skeletal tissues, Dot1l modulates osteogenesis by regulating cell cycle progression and key signaling pathways [21, 22]. The Notch signaling pathway plays dual roles in osteogenesis [23, 24], and its downstream target Chac1 (a glutathione-degrading enzyme) may negatively regulate osteogenic differentiation through its involvement in Notch signaling [25]. However, their key biological roles and potential crosstalk in maxillofacial bone repair have yet to be systematically elucidated, especially regarding periosteum-mediated regeneration.

In the present study, the activation of PDSCs is a key step in the process of bone regeneration, as it promotes bone tissue repair and regeneration. This study will further investigate the activation of PDSCs, and their osteogenic differentiation ability, and mechanism involved Dot1l/Chac1/Notch axis, providing a theoretical basis and identifying important targets for maxillofacial bone regeneration.

## 2. Methods

### 2.1. Mandibular Defect Model Construction

Ten 12-week-old adult male rats (300–350 g) were randomly divided into two groups: Sham operation group (*n* = 6) and periosteum-preserved mandibular defect model group (*n* = 6). First, the rats were anesthetized by inhaling 2 vol% isoflurane (R510-22-10, RWD). Next, the rats were fixed on the operating table to keep the mandibular part exposed. After disinfection with iodine, a 20 mm skin incision was made with a scalpel to separate the masseter and the pterygoid muscles, separated the periosteum while keeping it intact, and exposed the mandibular angle. Then, hemostasis of the superficial veins were achieved via electrocautery, and the segment of 5 mm × 5 mm × 3 mm of the mandible was excised with an oscillating saw. Finally, the subcutaneous tissue and periosteum were sutured with absorbable sutures, and the skin was sutured with nonabsorbable sutures. Postoperatively, the rats were given buprenorphine (0.1 mg/kg, once every 8 h, Simbadol, USA) and meloxicam (5 mg/mL, 1 mg/kg subcutaneously, once every 24 h for the first week, HY-B0261, MCE). The experiment lasted for 8 weeks, and the healing process of bone was observed by Hiscan XM Micro CT (Suzhou Hiscan Information Technology Co., Ltd). The X-ray tube settings were 80 kV and 100 μA, and images were acquired at 25 μm resolution. A 0.5° rotation step through a 360°angular range with 50 ms exposure per step was used. The images were reconstructed with Hiscan Reconstruct software (Version 3.0, Suzhou Hiscan Information Technology Co., Ltd) and analyzed with Hiscan Analyzer software (Version 3.0, Suzhou Hiscan Information Technology Co., Ltd). After the experiment, all the animals were euthanized. This retrospective study was approved by the Independent Ethics Committee of Shanghai Ninth People's Hospital, Shanghai Jiaotong University School of Medicine.

### 2.2. Isolation and Identification of Periosteal Stem Cells

The periosteal tissue of rat was separated using a blade and digested with collagenase P (1 mg/1mL, HY-E70005A, MCE) and 2 mg/mL dispase II (HY-131577, MCE) at 37°C for 1 h. The supernatant was removed by centrifugation, and excess DNA was digested by DNase I. Then, the mixture was filtered through a 70 μm filter to remove cell clumps and undigested tissue, and the cells were collected after centrifugation. The obtained cells were cultured in *α*-MEM medium containing 10% fetal bovine serum (FBS) at 37°C and 5% CO_2_. After culturing the cells for 3 days, the morphology of PDSCs was observed under a microscope (DM IL LED, Leica). For the identification of PDSCs cells, the cells were washed and incubated with CD44 antibody (15675-1-AP, Proteintech), CD90 antibody (66766-1-Ig, Proteintech), CD3 antibody (17617-1-AP, Proteintech), and CD45 antibody (20103-1-AP, Proteintech) to label PDSCs cells (CD44+CD90+CD3-CD45-). Finally, the data were processed using Flowjo software (v10, BD bioscience).

### 2.3. Lentivirus Transfection

Briefly, 4-week-old Sprague Dawley rats were euthanized and periosteum-derived PDSCs were obtained as above described. The pLV-CMV- 3×FLAG-Puro lentivirus vector containing Dot1l Ctrl/overexpression (OE) sequence or Chacl Ctrl/OE sequence were purchased from GenePharma Company (Shanghai, China) and tranfected into 293T cells, and then the 1 × 10^9^ lentiviral particle was obtained and infected into periosteum-isolated PDSCs with polybrene (2 μg/mL, HY-112735, MCE) to achieve overexpression of Dot1l or Chac1 in PDSCs. Next, Dot1l and Chac1 positive cells were screened by puromycin (1 mg/mL).

### 2.4. RNA-Seq

Total RNA were isolated using MJzol Animal RNA Isolation Kit (Majorivd), and purified using RNAClean XP Kit (Beckman Coulter). Then, the amount and purity of RNA was estimated with NanoDrop ND-2000 spectrophotometer (Thermo Fisher Scientific). Briefly, cDNA sequencing was performed using Illumina NovaSeq6000 sequencing platform, and the sequencing quality was determined by Q20 and Q30. After that, the raw sequencing data was analyzed by removing low-quality bases, comparing with the reference genome, and quantifying gene expression. For analysis of differential RNA expression, the edgeR software was used to perform the differential gene analysis between samples, and *p*-value was obtained. In addition, the differential expression multiple was calculated by FPKM value, that is, fold-change. Among them, genes that met the requirements, with *p* < 0.05 and log foldchange >1, were considered differential genes and were displayed in the form of volcano plots. In addition, Gene Ontology (GO) and Kyoto Encyclopedia of Genes and Genomes (KEGG) analyses are used to annotate the functions of differentially expressed genes and identify enriched biological pathways.

### 2.5. RT-qPCR Assay

Total RNA was extracted and then reversed into cDNA. The qRT-PCR was performed using a kit (RR420A, TAKARA) with specific primers: kmt2a, Tet3, Setd1b, Dot1l, RUNX2, OSX, ALP, OCN, Chac1, and Psen2. The expression of related genes was analyzed by 2^−ΔΔCt^, and the expression of GAPDH was used as control. The corresponding primers are shown in Table 1.

### 2.6. Western Blot Assay

Proteins were extracted, quantified, separated, and then transferred to polyvinylidene fluoride (PVDF) membranes (ISEQ00010, MerckMillipore). After then, the membranes were washed, blocked, and incubated with primary antibodies against Dot1l (90878S, CST), Chac1 (15207-1-AP, Proteintech), followed by horseradish peroxidase-conjugated secondary antibodies (SA00001-2, Proteintech). Finally, the protein bands were detected with enhanced chemiluminescence reagent, and the relative protein expression was all normalized to that of GAPDH. Uncropped western blot images are available in Figure S1.

### 2.7. ALP Staining, ARS Staining, and Oil Red Staining

ALP activity and mineralization nodules are well-established indicators in hard tissue regeneration studies [26, 27]. For ALP staining of PDSCs, cells were cultured in complete media, and when the cells were fuzed to 80%–90%, the medium was replaced with osteogenic induction medium containing including 10 mM β-glycerophosphate, 100 nM dexamethasone, and 50 µM ascorbate 2-phosphate. After 14 days, the cells were fixed with 4% paraformaldehyde for 20 min, and washed for ALP staining by ALP staining kit (E1041, Applygen). In addition, for ARS staining to reveal calcium deposition, cells were stained with alizarin red for 3 h using a kit (TMS-008, Sigma-Aldrich). Finally, the corresponding results were observed and imaged under a light microscope. For oil red O staining, PDSC cells were subjected to adipogenic induction differentiation, washed by PBS, and then stained by a commercial assay kit (RAXMX-90021, Cyagen).

### 2.8. Statistical Analysis

All statistical analyses were conducted using GraphPad Prism8. The statistical significance of independent experimental replicates in the graphs was determined using Student's *t*-test, one-way ANOVA and two-way ANOVA. Specific statistical analysis methods are marked in figure legends. *⁣*^*∗*^*p* < 0.05*⁣*^*∗∗∗*^/*⁣*^*∗∗∗∗*^*p* < 0.001 was accepted as statistically significant, ns was considered as not significant.

## 3. Results

### 3.1. Periosteum Preservation Promotes Jaw Bone Regeneration

**Figure 1:**
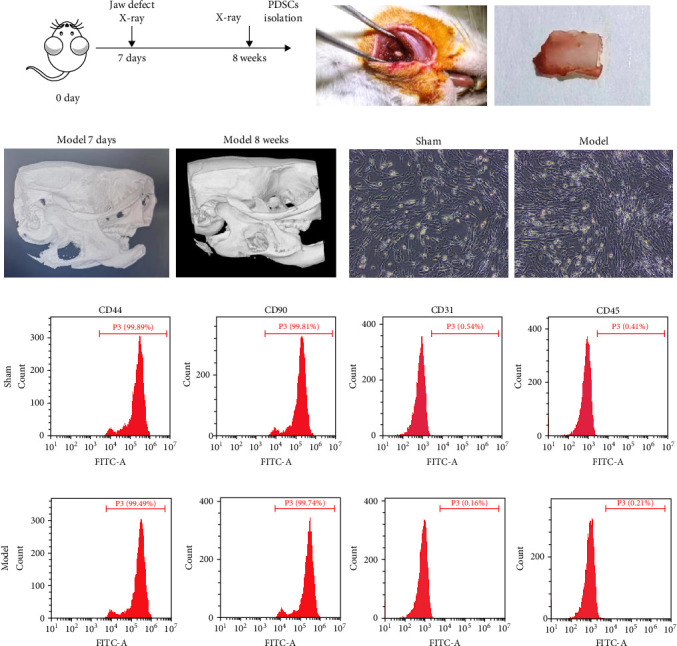
Mandibular defect repair and periosteal PDSCs isolation: (A) animal experimental design, (B) surgical image of a rat model of jaw defect with periosteum preserved, (C) X-ray images of the defect area in rats at day 7 and 8 weeks later, (D) morphologic photographs of periosteum-derived stem cells in sham and model rats, (E) the proportions of CD44+, CD90+, CD31+, and CD45+ cells in PDSCs isolated from the jaws of sham-operated rats were analyzed using flow cytometry, and (F) the proportion of CD44+, CD90+, CD31+, and CD45+ cells in PDSCs isolated from jaws of model rats were analyzed using flow cytometry.

We established a rat model of mandibular defect with preserved periosteum and observed the effect of bone repair by X-ray at 8 weeks after surgery. The results showed that the mandibular defect of rat with periosteum preserved was repaired after 8 weeks, suggesting that periosteum plays an important role in bone formation (Figure 1A–C). Next, we isolated PDSCs from the model group and sham group, which exhibited a spindle-shaped cell morphology (Figure 1D). Furthermore, flow cytometry analysis revealed that PDSCs isolated from the jaws of both sham-operated and model rats were positive for stem cell markers CD90 and CD44, but negative for CD45 and CD31 (Figure 1E,F). These results demonstrate that the preservation of periosteum critically promotes mandibular defect repair, and PDSCs exhibit characteristic stem cell properties, suggesting their potential role in periosteum-mediated bone regeneration.

### 3.2. Dot1l Expression is Downregulated During Mandibular Bone Regeneration

**Figure 2:**
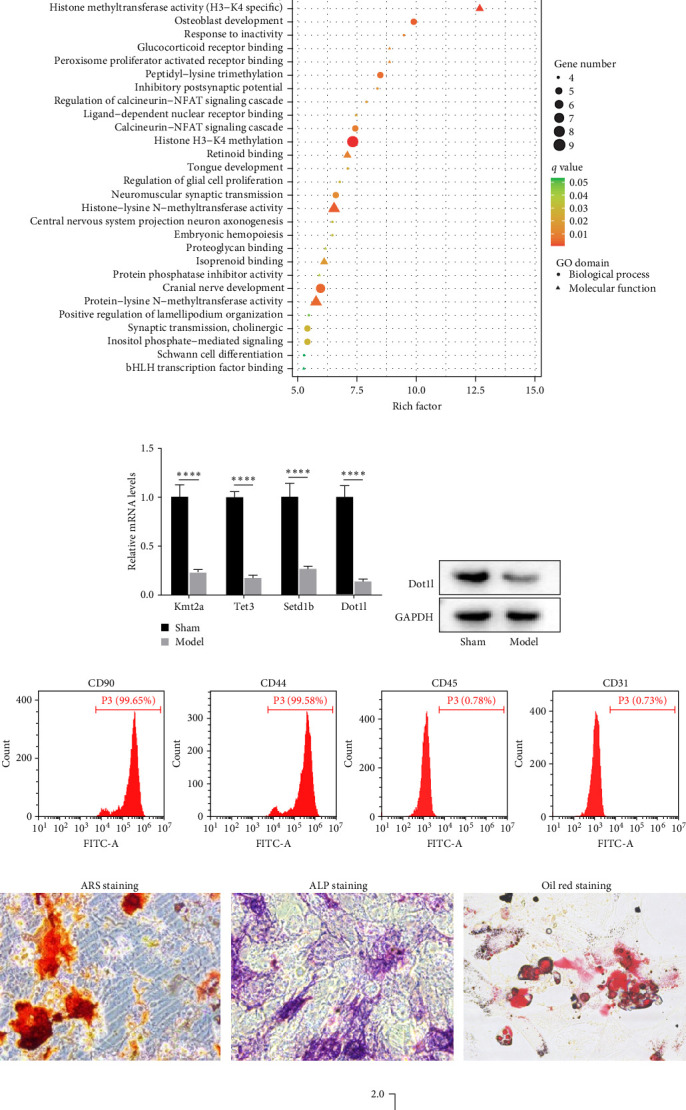
Expression pattern of Dot1l during mandibular defect healing, and Dot1l is important for the osteogenesis of PDSCs. (A) The volcano plot showed the differential genes in PDSCs isolated from the sham operation group and the periosteum-preserved jaw defect model group. (B) GO functional enrichment analysis showing the top 30 significantly enriched biological functions and roles. (C) The mRNA expression level of differential genes was detected using RT-qPCR assay, data were analyzed by using one-way ANOVA, with the Tukey's post hoc test. *⁣*^*∗∗∗∗*^*p* < 0.001. (D) The protein expression level of Dot1l was detected using western blotting assay. (E) The proportion of CD44, CD90, CD31, and CD45 positive expression cells in PDSCs from 4-week-old SD rats was analyzed by flow cytometry. (F) ARS staining was used to detect calcium deposition in PDSCs cells, ALP staining was used to detect ALP activity of PDSCs cells, oil red O staining was used to detect the lipogenic ability of PDSCs cells. (G) Detection of the Dot1l mRNA in PDSCs treated with Dot1l ctrl/overexpression via RT-qPCR assay, data were analyzed by *t*-test analysis, *⁣*^*∗∗∗*^*p* < 0.001. (H) Detection of the Dot1l mRNA in PDSCs treated with Dot1l ctrl/overexpression via western blot assay. (I) Detection of the mRNA expressions of osteogenic differentiation related genes in PDSCs treated with Dot1l ctrl/overexpression via RT-qPCR assay, data were analyzed by using one-way ANOVA, with the Tukey's post hoc test. *⁣*^*∗∗∗∗*^*p* < 0.001. (J) ALP staining was used to detect ALP activity of PDSCs cells treated with Dot1l ctrl/overexpression. (K) ARS staining was used to detect calcium deposition of PDSCs cells treated with Dot1l ctrl/overexpression.

Furthermore, to investigate the osteogenic mechanism of PDSCs, RNA sequencing was performed on PDSCs from the above two groups. The corresponding volcano map showed 669 differentially expressed genes between the two groups (Figure 2A). Meanwhile, the analysis of the top 30 GO terms indicates significant enrichment of genes associated with histone H3-K4 methylation (Figure 2B). The genes involved include Kmt2b, Kmt2e, Tet3, Setd1a, Tet2, Setd1b, Kmt2a, Ncoa6, Ash1l, Nsd1, and Dot1l. Based on the fold-change of downregulated expression, Kmt2a, Tet3, Setd1b, and Dot1l were selected for validation using RT-qPCR. Next, RT-qPCR assay was conducted to verify the top four differentially expressed genes (kmt2a, Tet3, Setd1b, and Dot1l), and the results showed that the mRNA expression level of Dot1l was significantly decreased in the model group (Figure 2C). Western blot experiment verified that the protein expression of Dot1l was also significantly decreased (Figure 2D). These results imply that Dot1l downregulation in PDSCs plays a critical role in mandibular bone regeneration.

### 3.3. Dot1l Overexpression Inhibited the Osteogenesis of PDSCs

To explore the effect of Dot1l on the osteogenesis of PDSCs, PDSCs were isolated from the periosteum of the jaws of 4-week-old rats. Flow cytometry showed that the proportions of cells expressing CD90 and CD44 were 99.65% and 99.58%, and the proportions of cells expressing CD45 and CD31 were 0.78% and 0.73%, respectively (Figure 2E). Furthermore, ARS, ALP, and oil red O staining showed that PDSCs had the osteogenic and lipogenic differentiation potential (Figure 2F). After that, we constructed Dot1l overexpressing PDSCs cells. According to RT-qPCR and western blots experiments, Dot1l OE increased the expression of Dot1l mRNA and protein in PDSCs (Figure 2 G,H). Further, the experimental results showed that the overexpression of Dot1l decreased the expression of osteogenic genes (RUNX2, OSX, ALP, and OCN) (Figure 2I), inhibited the ALP activity and mineralization deposits of PDSCs, demonstrating its ability to inhibit osteogenic differentiation (Figure 2J,K). These findings indicate that Dot1l acts as a negative regulator of osteogenic differentiation in PDSCs.

### 3.4. Dot1l Inhibited PDSCs Osteogenesis by Downregulating Chac1

**Figure 3:**
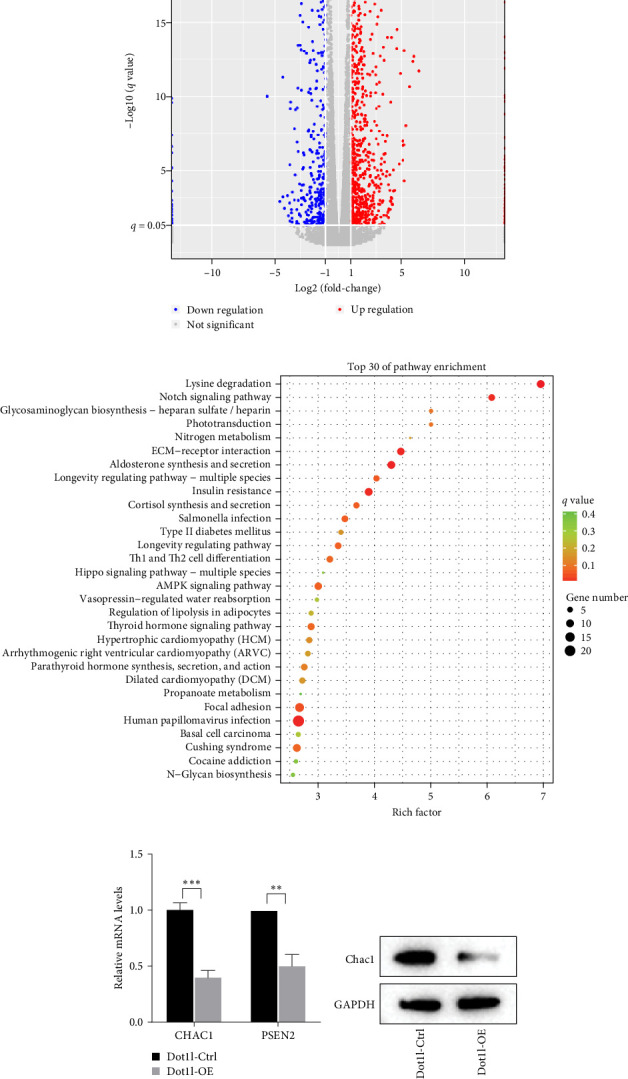
Dot1l targets Chac1 expression. (A) The volcano plot show differential genes in DOT1l-controlled/overexpressed PDSCs. (B) KEGG functional enrichment analysis showing the top 30 significantly enriched KEGG signaling pathways in the sham and model groups. (C) Detection of the mRNA expressions of differential genes(PSEN2 and CHAC1)in PDSCs treated with Dot1l ctrl/overexpression via RT-qPCR assay, data were analyzed by using one-way ANOVA, with the Tukey's post hoc test. *⁣*^*∗∗*^*p* < 0.01, *⁣*^*∗∗∗*^*p* < 0.001. (D) Detection of Dot1l protein expression in PDSCs treated with Dot1l ctrl/overexpression via western blotting assay.

**Figure 4:**
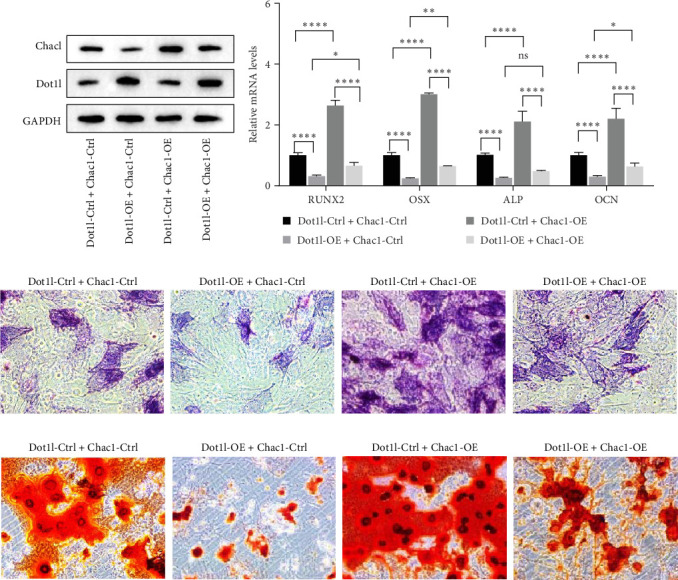
Dot1l regulates osteogenesis via Chac1. (A) Western blotting detected the expressions of Dot1l and Chac1 proteins in PDSCs transfected with Dot1l ctrl/OE + Chac1 Ctrl/OE lentivirus. (B) RT-qPCR assay detected the mRNA expression of osteogenic differentiation related genes in PDSCs transfected with Dot1l ctrl/OE + Chac1 Ctrl/OE lentivirus, data were analyzed by using two-way ANOVA, with the Tukey's post hoc test. *⁣*^*∗*^*p* < 0.05, *⁣*^*∗∗*^*p* < 0.01, *⁣*^*∗∗∗*^/*⁣*^*∗∗∗∗*^*p* < 0.001. (C) ALP staining was used to detect ALP activity of PDSCs cells infected with Dot1l ctrl/OE + Chac1 Ctrl/OE lentivirus. (D) ARS staining was used to detect calcium deposition in PDSCs cells infected with Dot1l ctrl/OE + Chac1 Ctrl/OE lentivirus.

To further explore the specific mechanism by which Dot1l regulates the osteogenic differentiation of mandibular periosteal stem cells, we conducted RNA-seq analysis to identify the target genes affected by Dot1l. Based on the screening criteria, the sequencing results of the control group and the overexpression group revealed 552 differentially expressed genes (Figure 3A). According to existing studies, the Notch signaling pathway has been widely confirmed as a key regulator of osteogenic differentiation [28]. Notably, KEGG enrichment analysis of the top 30 differentially expressed genes between the sham and model groups in this study revealed significant differences in the Notch signaling pathway. Therefore, it is suggested that mandibular defects in this study may regulate spontaneous mandibular regeneration via the Notch pathway (Figure 3B). As expected, we found that the Notch signaling pathway (GO: 0007219) exhibited significant differences in the Dot1l OE vs. Dot1l Ctrl comparison, and Chac1 and PSEN2 may play roles in this pathway. Further analysis using RT-qPCR revealed that Chac1 and PSEN2 were all downregulated in the overexpression group, with Chac1 showing the most significant decrease in expression levels (Figure 3C). Western blot experiment verified that the protein expression of Chac1 was also significantly decreased in Dot1l OE group (Figure 3D). Based on this, we subsequently constructed an overexpression lentivirus for Chac1 and performed a Dot1l OE and Chac1 OE double transfection rescue functional experiment. First, the results of the western blot experiment showed that the double transfection of Dot1l OE and Chac1 OE significantly increased the protein expression of Dot1l and Chac1, confirming the successful transfection of PDSCs (Figure 4A). Additionally, functional experiments showed that Chac1 overexpression significantly reversed the inhibitory effects of Dot1l overexpression on the expression of osteogenic-related genes (RUNX2, OSX, ALP, and OCN) (Figure 4B), as well as on ALP activity and calcium deposition (Figure 4C,D). These findings demonstrate that Dot1l overexpression inhibits PDSC osteogenic differentiation by downregulating Chac1 expression. Together, these data reveal that the Dot1l-Chac1 pathway negatively controls osteogenesis in PDSCs.

## 4. Discussion

Recent studies have confirmed that the periosteum plays a crucial role in bone repair [29]. Various bionic periosteum substitutes (such as small intestinal submucosal bionic periosteum and biodegradable polymer membranes) contribute to bone regeneration [30]. In addition to these, drug stimulation and transcutaneous electrical nerve stimulation also play important roles in promoting bone regeneration [31, 32]. The results of this study showed that PDSCs in the bone defect model group with periosteal preservation exhibited stronger spontaneous bone regeneration ability. In contrast, the regenerative ability of PDSCs in the sham operation group may be limited, possibly because the PDSCs in the model group were more stimulated during the bone defect repair process, while the cells in the sham operation group remained in a static state. As described in previous clinical studies, periosteum-preserving mandibular resection can promote spontaneous bone healing in young or pediatric patients with osteonecrosis, and even in older patients when periosteum is preserved during surgery [16, 29, 33]. This further indicates that mechanical stress following bone removal can drive biochemical signaling in PDSCs, subsequently regulating various aspects of cell behavior and participating in differentiation and growth [34].

Stem cells are the cornerstone of bone repair. Numerous studies have focused on bone marrow-derived mesenchymal stem cells (BMSCs), confirming that when stimulated by immune cells and various cytokines, BMSCs can promote blood vessel aggregation and bone repair following a bone defect [35–37]. Periosteal-derived stem cells (PDSCs) are located in the inner periosteal layer and exhibit MSC markers as well as multidirectional differentiation potential [10]. When bone defects occur, PDSCs are activated, recruited, proliferated, and differentiated, playing a key role in endogenous bone repair. However, the role of these cells has not been widely acknowledged [35]. Recent studies have confirmed that PDSCs exhibit stronger growth and differentiation activity compared to BMSCs. Therefore, it is essential to further explore the functions and biological mechanisms of PDSCs, which could provide new perspectives and targets for bone tissue engineering cell therapy [11]. Thus, this further supports our RNA-seq sequencing of isolated periosteal stem cells.

Importantly, histone H3-K4 methylation was significantly enriched in GO pathway analysis, with H3K79 methyltransferase Dot1l being identified as highly sensitive to bone defect stimulation for the first time. Notably, Dot1l levels were found to be downregulated in activated PDSCs, suggesting a potential regulatory role in the response to bone injury. Dot1l is a key histone methylation regulator involved in transcriptional regulation. Previous studies have reported that Dot1l activity is significantly reduced in areas of cartilage injury, and that its expression in chondrocytes is associated with cartilage health and endochondral bone formation [20, 21]. In addition, the role of Dot1l in inhibiting osteoclast activity and preventing osteoporosis has been reported, but its regulatory role in osteogenesis remains unclear [38]. Our data indicate that the expression of Dot1l is significantly correlated with osteogenesis in PDSCs. Overexpression of Dot1l inhibited ALP activity, calcium deposition, and the expression of osteoblast-related genes in PDSCs, suggesting that Dot1l plays an indispensable role in jaw regeneration during the healing process. The KEGG enrichment analysis of the top 30 differentially expressed genes between the sham and model groups revealed a significant involvement of the Notch signaling pathway. This suggests that the observed mandibular defect in our study may regulate spontaneous mandibular regeneration through modulation of the Notch pathway. These findings highlight the potential of targeting the Notch pathway in understanding and promoting mandibular regeneration. Indeed, previous studies have also suggested that Dot1l can indirectly activate Notch signaling, influencing the progression of various diseases [39, 40].

Furthermore, through RNA-seq analysis, the Notch signaling pathway (GO: 0007219) exhibited significant differences. Specifically, two genes:Psen2 and Chac1 were found to be significantly downregulated in Dot1l-overexpressing PDSCs, suggesting a potential link between Dot1l expression and the regulation of Notch signaling components. According to existing studies [41], the upregulation of Psen2 is positively correlated with the osteogenic differentiation marker protein (OCN). Fortunately, Chac1 showed the most significant differential expression level. It is well known that the Notch pathway has played an important role in osteogenic differentiation and bone development in recent years [42]. Chac1 is a molecule associated with the Notch pathway. According to KEGG pathway analysis, Chac1, as a negative regulatory gene in the Notch signaling pathway, may be related to osteogenic differentiation [25]. Currently, there are no reports on the relationship between Chac1 and the osteogenic differentiation of PDSCs, and this connection remains to be further explored. The mechanistic and functional studies in this research have found that Dot1l can inhibit Chac1, thereby exerting an inhibitory effect on the osteogenic differentiation of PDSCs. This further confirms that Dot1l may be a key upstream molecule regulating Chac1 in osteogenic activation and could serve as a promising therapeutic target for promoting the spontaneous healing of the jaw in PDSCs. However, this study has some limitations. The specific changes in the Notch signaling pathway were not explored in detail, and further research is needed to investigate how Notch signaling interacts with the Dot1l/Chac1 axis in the context of bone regeneration. Additionally, the findings are primarily based on in vitro experiments, and their relevance in vivo remains uncertain. Further studies using animal models are necessary to validate the therapeutic potential of targeting Dot1l for bone regeneration.

## 5. Conclusion

In conclusion, Dot1l overexpression downregulates Chac1, thereby inhibiting the osteogenic activity of PDSCs through Notch, potentially through the regulation of the Notch signaling pathway. Therefore, targeting the Dot1l/Chac1 axis could help accelerate spontaneous jaw regeneration, and serve as a potential target for promoting bone repair.

## Figures and Tables

**Table 1:** The primer information for the genes used in the RT-qPCR experiment.

Genes	Primers	Sequences (5'-3')	Product size (bp)
*Dot1l*	Sense	ACAGCAGGAACTTGAGTGACATTGG	87
	Antisense	GCTTGCCAGTCCATGACACAGAG	

*Chac1*	Sense	CTTGCTTGCCGAGGCTTCTCTG	105
	Antisense	GGCTTCCAGGTGCTCATCTTGTG	

*kmt2a*	Sense	TGACAGGAGAGGCAGACGGTATTC	119
	Antisense	GTGGAGGTGGCGAGGAGGAG	

*Tet3*	Sense	GGTGGAAGAGGACGAGGAGGAG	145
	Antisense	GAGTGGTGTGGTGGCATGTAGC	

*Setd1b*	Sense	TGTCCGTGTCCTCATCTTCCTCAG	125
	Antisense	CTCTTCCTCTTCCTCCTCCTCAGTG	

*RUNX2*	Sense	CTTCGTCAGCGTCCTATCAGTTCC	149
	Antisense	TCCATCAGCGTCAACACCATCATTC	

*OSX*	Sense	GCCTACTTACCCGTCTGACTTTGC	141
	Antisense	CCCTCCAGTTGCCCACTATTGC	

*ALP*	Sense	CACGGCGTCCATGAGCAGAAC	83
	Antisense	CAGGCACAGTGGTCAAGGTTGG	

*PSEN 2*	Sense	TCAGCCGAGAGCCCTACATCAC	115
	Antisense	GGTCCTCCTCACAGTCGTCCTC	

*OCN*	Sense	GGACCCTCTCTCTGCTCACTCTG	124
	Antisense	ACCTTACTGCCCTCCTGCTTGG	

## Data Availability

The data that support the findings of this study are available from the corresponding author upon reasonable request.

## Supporting Information

Supporting Information Figure S1: Western blot original images. The supporting information include the original western blot images corresponding to the protein expression analysis presented in the study.
