# Supplementary figures and images for: Dot1l Regulates the Spontaneous Bone Regeneration of Periosteum-Derived Stem Cells by Regulating Chac1 Expression
Source: Stem Cells Int. 2025 Jul 9;2025:1508850. doi: 10.1155/sci/1508850 (PMC12267974; doi:10.1155/sci/1508850)

Fig. 2D

Dot1l

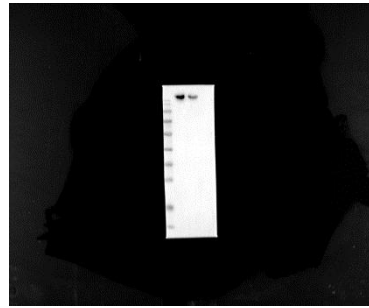

GAPDH

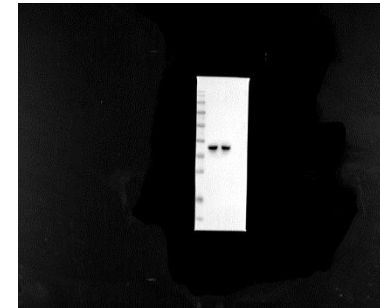

Fig. 2H

Dot1l

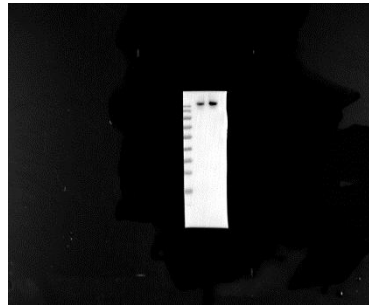

GAPDH

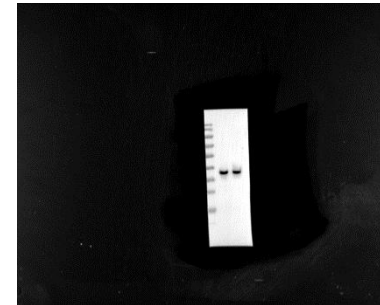

Fig. 3D

Chac1

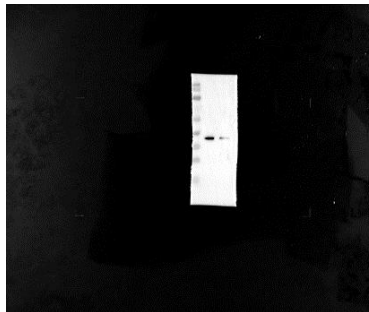

GAPDH

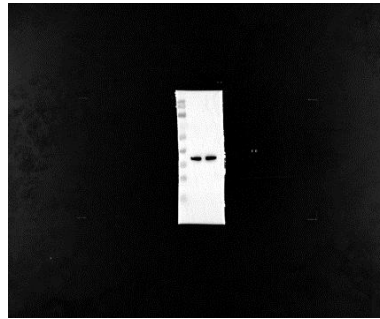

Fig. 4A

Chac1

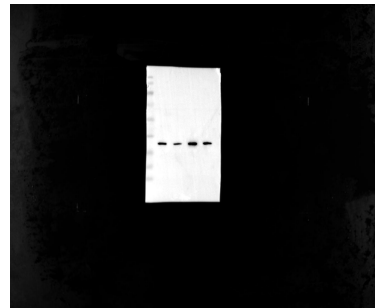

Dot1l

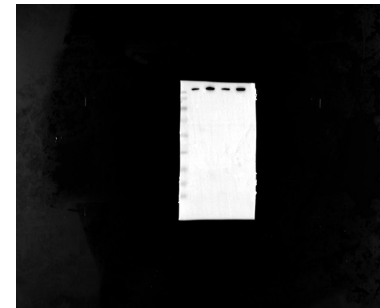

GAPDH

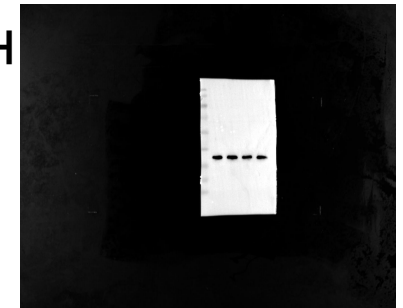

Supplement: Supporting Information — Figure S1: Western blot original images. The supporting information include the original western blot images corresponding to the protein expression analysis presented in the study. [file 1508850.f1.pdf]
